# Transcriptome analysis in switchgrass discloses ecotype difference in photosynthetic efficiency

**DOI:** 10.1186/s12864-016-3377-8

**Published:** 2016-12-16

**Authors:** Desalegn D. Serba, Srinivasa Rao Uppalapati, Nick Krom, Shreyartha Mukherjee, Yuhong Tang, Kirankumar S. Mysore, Malay C. Saha

**Affiliations:** 1 Forage Improvement Division, The Samuel Roberts Noble Foundation, 2510 Sam Noble Parkway, Ardmore, OK 73401 USA; 2 Plant Biology Division, The Samuel Roberts Noble Foundation, Ardmore, OK 73401 USA; 3 Computing Services, The Samuel Roberts Noble Foundation, Ardmore, OK 73401 USA; 4 Present Address: Agricultural Research Center-Hays, Kansas State University, 1232 240th Avenue, Hays, KS 67601 USA; 5 Present Address: DuPont Crop Protection, Stine-Haskell Research Center, Newark, DE 19711 USA; 6 Present Address: Syngenta, Stanton, MN 55018 USA

**Keywords:** Switchgrass, Transcriptome, Ecotype, Photosynthesis, SNP markers

## Abstract

**Background:**

Switchgrass, a warm-season perennial grass studied as a potential dedicated biofuel feedstock, is classified into two main taxa – lowland and upland ecotypes – that differ in morphology and habitat of adaptation. But there is limited information on their inherent molecular variations.

**Results:**

Transcriptome analysis by RNA-sequencing (RNA-Seq) was conducted for lowland and upland ecotypes to document their gene expression variations. Mapping of transcriptome to the reference genome (*Panicum virgatum* v1.1) revealed that the lowland and upland ecotypes differ substantially in sets of genes transcribed as well as levels of expression. Differential gene expression analysis exhibited that transcripts related to photosynthesis efficiency and development and photosystem reaction center subunits were upregulated in lowlands compared to upland genotype. On the other hand, catalase isozymes, helix-loop-helix, late embryogenesis abundant group I, photosulfokinases, and S-adenosyl methionine synthase gene transcripts were upregulated in the upland compared to the lowlands. At ≥100x coverage and ≥5% minor allele frequency, a total of 25,894 and 16,979 single nucleotide polymorphism (SNP) markers were discovered for VS16 (upland ecotype) and K5 (lowland ecotype) against the reference genome. The allele combination of the SNPs revealed that the transition mutations are more prevalent than the transversion mutations.

**Conclusions:**

The gene ontology (GO) analysis of the transcriptome indicated lowland ecotype had significantly higher representation for cellular components associated with photosynthesis machinery controlling carbon fixation. In addition, using the transcriptome data, SNP markers were detected, which were distributed throughout the genome. The differentially expressed genes and SNP markers detected in this study would be useful resources for traits mapping and gene transfer across ecotypes in switchgrass breeding for increased biomass yield for biofuel conversion.

**Electronic supplementary material:**

The online version of this article (doi:10.1186/s12864-016-3377-8) contains supplementary material, which is available to authorized users.

## Background

Increasing emission of carbon dioxide into the atmosphere from burning nonrenewable fossil fuels causes the earth to warm up by trapping more heat from the atmosphere. A bioenergy alternative to the continued consumption of nonrenewable fossil fuels will avert serious environmental, social and economic concerns for the future. Thus, finding renewable alternative fuel sources that are environmentally friendly and economically feasible will achieve a dual goal of improving energy security and decreasing greenhouse gases emissions emitted from the transportation sector and industry [1]. An increased attention is given to the lignocellulosic feedstocks as an alternative [2] to starch- and sucrose based feedstocks that are in competition as food crops [3].

Switchgrass (*Panicum virgatum* L.), a warm-season 4-carbon (C4) fixation perennial grass native to North America, is being developed as source of dedicated biofuel feedstock for production of transportation fuel [4]. This choice is attributed to several features of switchgrass such as high biomass yield potential, low external input requirements, and agronomic performance on marginal lands that are too dry and infertile for most other agriculture crops.

Switchgrass is a genetically and morphologically diverse species with an array of ploidy levels and classified into two phenotypically distinct ecotypes: lowland and upland [5]. This classification is based on strong ecotypic adaptation difference and population structure across the continental range [6], which is based on their ploidy level and morphological variations [7]. The lowland ecotypes are generally found in warmer and wetter areas of the southern United States. Morphologically, the lowland ecotypes are taller with thicker stems and wider leaves than upland ecotypes. The upland ecotypes are generally smaller in size, have narrower stems and leaves, and produce less biomass than the lowland ecotype. But they are adapted to the dry areas and are capable of overwintering in colder climates of the northern United States.

The causes of genetic diversity in natural populations and the relative influences of ecology versus population history are still largely unknown [8]. A switchgrass synthetic cultivar was developed from upland (Summer) into lowland (Kanlow) cross and released in the midwestern United States for its better winter survival than the lowland-type and higher biomass yield potential than the upland-type [9]. This development of a heterotic cultivar from the upland into lowland ecotype cross echoes the natural process of inter-ecotype breeding for better adaptation. This population can also be used to select genotypes of better biomass yield potential than upland-type and reduce recalcitrance for better biofuel conversion.

The other conspicuous difference between the two switchgrass ecotypes is in the length of vegetative growth; the lowlands regrow early in the spring and flower late in the summer compared to the uplands, which generally have a shorter vegetative growth period as a result of late regrowth coupled with early flowering in the Southern Great Plains (Serba et al., unpublished). However, intraspecific comparative genome analyses revealed that the lowland and upland ecotypes are completely collinear and have similar recombination rates [10]. They intercross freely at same ploidy levels and produce fertile progenies, thus there is free gene flow in both directions. Based on the number of marker loci mapped in the lowland genotype AP13 and the upland genotype VS16, lower level of genome heterozygosity was speculated for the uplands than the lowlands.

The amount of variation observed between DNA sequences from distinct genotypes of a given species is a reflection of genetic diversity [11]. Analyzing the transcribed portions of the genome is an economical approach for plants with large genome sizes like switchgrass. The functional information derived from the analysis of ESTs can be used for the development of molecular markers, comparative genomics, genetic analysis of adaptive traits, and gene discovery [12]. Gene expression variation between tissues or cell types [12], developmental stages [13], genotypes [14], and for differences in stress tolerance [15] have been documented in switchgrass. In addition to the nuclear genome, chloroplast genome sequence variation was also reported for the lowland and upland ecotypes. Ecotype comparison of the chloroplast genomes revealed a total of 224 bp of insertions and deletions that the chloroplast sequence of lowland ecotype Kanlow (K5) is 58 bp overall longer than the upland ecotype Summer with polymorphic rates (0.05% for single nucleotide polymorphisms and 0.02% for insertions or deletions) between the ecotypes [16]. Similar levels of intersubspecific polymorphic rates were reported between chloroplast genomes of rice, *Oryza indica* and *O. japonica* [17]. On the other hand, 59 bp deletion in trnL (UAA) intron sequences of lowland ecotype AP13 chloroplast genome was observed compared to the upland ecotype VS16 [18]. A *Bam*HI RFLP polymorphism in *RbcL* gene present in upland and absent in lowland cultivars was also reported [19]. This polymorphism information between upland and lowland ecotypes would facilitate a study of cultivar diversity, improved analyses of population structure, direction of gene flow and genetic mapping [16].

Apart from the distinct phenotypic and chloroplast genome differences, variation in gene expression patterns between lowland and upland ecotypes has not been thoroughly investigated yet. RNA-Seq analysis is reported as the most effective strategy that can be used to discover new genes as well as to provide high-density markers [20]. In this study we assessed the differential gene expression profiles of upland and lowland ecotypes in tetraploid representative genotypes using RNA-Seq and examined the ESTs for development of molecular markers useful for mapping agronomic and adaptive traits in switchgrass. The outcome of this investigation in addition to broadening our understanding of the ecotypic differences in switchgrass, it can be applied in molecular breeding to fast-track the development of improved cultivars for yield and quality for biofuel conversion. The RNA-Seq data is also used to better understand the transcriptomic differences that are reflected in the morphological and ecological adaptation of the lowland and upland ecotypes, especially during active growth stages. The high-throughput markers developed in this study will also facilitate the accurate and efficient discrimination of the heterogeneous switchgrass gene pools [16].

## Results and Discussion

### RNA-Seq data acquisition and reads mapping to switchgrass reference genome

Transcriptome of switchgrass ecotypes was profiled using two lowland genotypes (AP13 and K5) and an upland genotype (VS16) that are tetraploids (2n = 4x = 36). A high-throughput RNA-Seq data set was generated for these three genotypes that provide transcriptome analysis through quantitative readouts [21]. A total of 265.2 million 100 bp reads were obtained for the three genotypes from three biological replicates. Out of the 265.2 million reads, 209.7 million quality reads were aligned to the reference switchgrass genome sequence, *P. virgatum* v1.1 (http://www.phytozome.net; accessed 30 November 2015). These reads were then used for a reference-guided assembly and differential expression analysis.

**Fig. 1:**
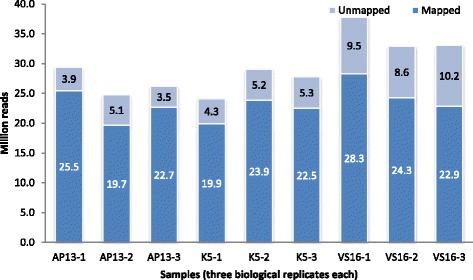
Total mapped and unmapped RNA-Seq clean reads for the three genotypes representing lowland (AP13 and K5) and upland (VS16) switchgrass ecotypes

The total number of reads obtained were significantly higher in VS16 (103.8 million) than in AP13 (80.3 million) and K5 (81.1 million) (Fig. 1). A total of 84.6% (67.9/80.3) for AP13, 81.8% (66.3/81.1) for K5, and 72.7% (75.5/103.8) for VS16 were mapped to the reference genome. The result indicated that there was no significant difference (*p* < 0.01) among the three genotypes in the number of reads mapped to the reference genome. As increasing the number of replicate samples improves detection power over increased sequencing depth [22], three biological replicates for each genotype were used in this experiment for read mapping and downstream analysis such as assembly of gene transcripts and differential profiling. Analysis of variance among the biological replicates were not significant, which implies high repeatability of the samples (Additional file 1: Table S1). However, an average of 20.3% of the reads could not be mapped to the *P. virgatum* v1.1 switchgrass preliminary release reference genome, probably due to incomplete genome coverage of the reference sequence. As the number of mapped sequences were large enough to successfully assemble transcripts with reasonable depth and coverage, no separate *de novo* assembly was conducted for the unmapped sequences.

### Differentially expressed genes between genotypes and ecotypes

Mapping the quality trimmed pair-end reads of the AP13, K5 and VS16 genotypes representing the ecotypes of switchgrass enabled us to construct a total of 37,611 differentially expressed transcripts. The total number of differentially expressed transcripts (q-value ≤ 0.05) were 29,176 between AP13 and VS16, 17,863 between AP13 and K5, and 23,207 between K5 and VS16 (Additional file 2: Table S2). Among the largest number of differentially expressed transcripts (between AP13 and VS16), 22,761 were annotated genes and had homologs in either Arabidopsis or rice genome as determined by slimGO terms. The remaining 6,415 were not annotated as gene transcripts.

**Fig. 2:**
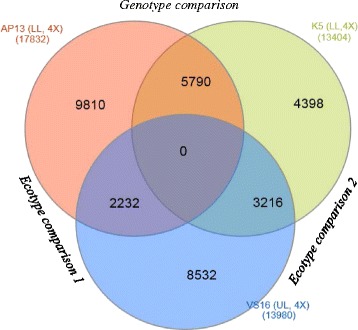
Comparisons of differential gene expression (upregulation) among two lowland (AP13 and K5) and an upland (VS16) switchgrass genotypes (two-fold change, q-value ≤ 0.05) constructed using Cufflinks v2.1.1. (LL = lowland ecotype, UP = upland ecotype, 4 × = tetraploid). The comparison reveals distinct ecotype differences as compared to two genotypes within the lowland ecotype

Three-way comparison of 17,832 in AP13, 13,404 in K5, and 13,980 in VS16 upregulated transcripts highlighted ecotype difference than genotype differences (Fig. 2). Ecotype difference in gene expression was apparent by the smaller number of commonly upregulated transcripts between the ecotypes (2,232 between AP13 and VS16, 3,216 between K5 and VS16) as compared to the 5,790 between AP13 and K5, the two genotypes of the lowland ecotype.

The expression level of the gene transcripts ranged from 0 to 9,116 FPKM (Fragments Per Kilobase of transcripts per Million mapped reads) in all the three genotypes. Among the total transcripts detected in the three genotypes, differential expression analysis also revealed that a total of 1,736 genes had no detection in AP13; similarly 2,308 and 2,131 had no detection in K5 and VS16, respectively. These under detected genes may also designate the ecotype variations due to gene expression level or high degree of sequence divergence, but their function has not yet been discerned.

### Function of differentially epxressed genes (DEGs)

We annotated the 29,176 transcripts by blasting all the distinct unigene sequences against PFAM, PANTHER, KOG, KEGG, and slimGO database (http://bioinfo.cau.edu.cn/agriGO) by BLASTX with a cut-off E-value of 10^−5^. Genes that were upregulated significantly (≥2-fold change, q ≤ 0.05) in the lowland ecotypes compared to the upland VS16 (Table 1) signifies potential ecotypic variation. Some of these genes had multiple copies tandemly distributed in the genome with the copy number ranging from 2 to 10 (Additional file 1: Table S1). The differential expression between the lowland and upland ecotypes was consistent for most of the genes across the gene copies.

**Table 1:** Photosynthetic efficiency related gene transcripts differentially upregulated in lowlands as compared to upland switchgrass with more than two-fold change (q-value ≤ 0.01)

No	Rice homolog	Protein function description	Average FPKM_ Lowland	Average FPKM_ VS16	Average Fold change
1	Peroxiredoxin	Antioxidant defense system	280	98	2.9
2	Peptidyl-prolyl cis-trans isomerase	Catalysis of the geometric or structural changes within one molecule	540	211	2.6
3	NAD dependent epimerase/dehydratase family protein	Catalytic activity and coenzyme binding	308	121	2.5
4	Glutathione S-transferase	Detoxication and toxification mechanisms	351	122	2.9
5	Ftsh protease	Eliminating abnormal membrane proteins in chloroplast	1005	401	2.5
6	AAA-type atpase family protein	F-type ATPase	308	68	4.8
7	ATP synthase B chain, chloroplast precursor	F-type ATPase	780	314	2.5
8	ATP synthase F1, delta subunit family protein	F-type ATPase	489	168	2.9
9	ATP-dependent Clp protease ATP-binding subunit clpa homolog, chloroplast precursor	F-type ATPase	690	336	2.1
10	Glyceraldehyde-3-phosphate dehydrogenase	Glycolysis	893	356	2.6
11	Cysteine proteinase 1 precursor	Hydrolysis of peptide bonds in a polypeptide chain	409	107	4.2
12	B-box zinc finger family protein	Intracellular protein transport	310	118	2.6
13	Chlorophyll A-B binding protein	LHC-Antana protein	2111	794	5.9
14	Zinc finger A20 and AN1 domain-containing stress-associated protein	Metal ion and DNA binding	347	139	2.5
15	Metallothionein	Metal ion binding	4704	1446	5.2
16	Metallothionein-like protein 3B	Metal ion binding	1471	94	14.3
17	NDH-M H Plastoquinine dehydrogenase	Photosynthetic electron transport	276	92	3.0
18	2Fe-2S iron-sulfur cluster binding domain containing protein	Photosynthetic electron transport	462	136	3.4
19	Calvin cycle protein CP12	Photosynthetic electron transport	1664	402	4.1
20	Carbonic anhydrase, chloroplast precursor	Photosynthetic electron transport	1229	300	4.5
21	Ferredoxin--NADP reductase, chloroplast precursor	Photosynthetic electron transport	523	210	2.6
22	Fructose-1,6-bisphosphatase	Photosynthetic electron transport	282	106	2.7
23	Fructose-bisphospate aldolase isozyme	Photosynthetic electron transport	1724	714	2.6
24	Phosphoglycerate kinase protein	Photosynthetic electron transport	312	106	3.1
25	Phosphoribulokinase/Uridine kinase family protein	Photosynthetic electron transport	285	72	4.2
26	Pyruvate, phosphate dikinase, chloroplast precursor	Photosynthetic electron transport	885	376	2.4
27	Ribulose bisphosphate carboxylase small chain, chloroplast precursor	Photosynthetic electron transport	2367	1113	2.2
28	Ribulose-phosphate 3-epimerase, chloroplast precursor	Photosynthetic electron transport	321	98	3.3
29	Thioredoxin	Photosynthetic electron transport	725	237	3.9
30	Transketolase, chloroplast precursor	Photosynthetic electron transport	399	135	3.2
31	Proton gradient regulation 5 (pgr5)	Photosynthetic electron transport in photosystem I	294	128	2.3
32	Photosystem I reaction center subunit, chloroplast precursor	Photosystem I subunits	892	416	2.2
33	Cytochrome b6-f complex iron-sulfur subunit, chloroplast precursor	Photosystem II main subunits	624	153	4.1
34	Cytochrome b6f complex subunit	Photosystem II main subunits	284	96	2.9
35	Oxygen evolving enhancer protein 3 domain containing protein	Photosystem II subunits	233	84	3.0
36	Photosystem II reaction center W protein, chloroplast precursor	Photosystem II subunits	555	176	3.6
37	Dehydrin	Plant response and adaptation to abiotic stresses	299	111	2.7
38	Fatty acid desaturase	Plant responses to abiotic stresses	263	102	2.6
39	Enzyme of the cupin superfamily protein	Protect plants from the effects of oxidative stress	447	205	2.2
40	Tetratrico peptide repeat region TPR domain protein	Protein binding	239	77	3.1
41	60S acidic ribosomal protein	Protein synthesis	281	114	2.5
42	Elongation factor	Protein synthesis in the process of cell cycle and elongation	274	82	3.6
43	Hypoxia-responsive family protein	regulation of growth and and developmnt	711	148	4.8
44	Glycine-rich protein A3	RNA-binding	351	158	2.2
45	RNA recognition motif containing protein	RNA-binding domain	905	311	3.0
46	BBTI8 - Bowman-Birk type bran trypsin inhibitor precursor	Serine-type endopeptidase inhibitor activity	316	95	3.6
47	Egg apparatus-1	Small secretory proteins and pollen tube guidance	227	63	4.4
48	Elongation factor thermo unstable (EF-Tu)	Synthesizes new proteins by translation at the ribosome	484	232	2.1
49	Oryzain gamma chain precursor	cysteine-type peptidase activity	546	148	3.6
50	Ubiquitin family protein	Targeted protein degradation	477	200	2.4
51	Ubiquitin-conjugating enzyme	Targets a protein for degradation via the proteasome	386	110	4.0
52	CCT/B-box zinc finger protein	Transcription factor	291	114	2.6
53	Ribosomal protein L35	Translation, ribosomal component	273	116	2.4
54	Aquaporin protein	Transport of water and small solutes	1022	239	4.8
55	Membrane protein	Water and nutrient transport across membranes	799	255	3.1

There was significant differential expression between AP13 and K5; upregulation of gene transcripts related to photosynthesis were the most significantly enriched GO categories amongst the DEGs between the ecotypes. Annotation of the putative genes that were upregulated in both the lowland ecotypes encode chloroplast precursors, photosynthetic electron transport system and associated ATP synthesis (Table 1). Some of these upregulated genes encode carbonic anhydrase, cytochrome b6-f complex iron-sulfur subunit, ferredoxin-NADP reductase, phosphoribulokinase, photosystem I reaction center subunit, photosystem II 10 kDa polypeptide, photosystem II reaction center W protein, pyruvate, phosphate dikinase, ribosomal protein L35, ribulose bisphosphate carboxylase, and transketolase. Genes encoding photosynthetic electron transport system (Additional file 3: Figure S1) were also among the upregulated group in the lowland ecotype. It is evident that many gene products encode the two main steps of photosynthesis [23], and the number of genes expressed can affect the inherent photosynthetic activity and environmental stress responses in different ecotypes.

Chlorophyll concentration is the main factor (apart from light intensity, carbon dioxide concentration, water availability and temperature) affecting the rate of photosynthesis reaction as it absorbs the light energy, without which the reactions cannot take place. Therefore, the expression of photosynthetic genes in the nucleus is influenced by the retrograde (from the chloroplast to the nucleus) signaling that utilizes photosynthetic electron transport and redox signaling [23]. The differential expression of photosynthesis-related genes specifically in the lowland-types suggests the exceedingly efficient light absorption and higher photosynthetic rate of this ecotype. Higher content of photoreceptor proteins and non-protein photo-pigments contribute to the higher light absorption efficiency and photosynthetic rate in the lowland ecotype than the upland ecotype.

In addition, genes involved in plant response and adaptation to stresses such as peroxiredoxin, glutathione S-transferase, dehydrin, fatty acid desaturase and hypoxia-responsive family protein were significantly upregulated in both the lowland genotypes compared to the upland genotype. As the plants were not subjected to any stress during the experiment, the high expression of such stress-responsive genes indicates that these proteins may have other functions in the plant growth and development that may reflect the difference between the switchgrass ecotypes. For instance, peroxiredoxin functions as a peroxidase to sense and regulate local peroxides [24] and has a vital role in antioxidant defense in photosynthesis, and respiration that is modulating redox signaling during development [25]. ATPases (adenosine triphosphatase) associated with diverse cellular activities and Calvin cycle protein chloroplast protein 12 (CP12) were significantly upregulated in the two lowland genotypes (AP13 and K5) than the upland VS16. The upregulation of ATPases indicate high dephosphorylation reaction in lowlands to release energy to facilitate other chemical reactions that would not otherwise take place. CP12 is a small, redox-sensitive protein involved in thioredoxin-mediated regulation of the key enzymes of the reductive pentose phosphate cycle (Calvin cycle) such as NAD(P)H-glyceraldehyde-3-phosphate dehydrogenase in response to changes in light intensity [26, 27].

On the contrary, catalases, heavy-metal-associated domain-containing protein, helix-loop-helix DNA-binding domain containing protein, late embryogenesis abundant group 1, protease inhibitors, methyltransferases, NADP-dependent oxidoreductase, and S-adenosylmethionine synthetase were upregulated in the upland VS16 compared to the lowland ecotype (Table 2). Transcriptional regulatory proteins such as helix-loop-helix DNA-binding domain containing protein may have likely roles in a wide array of developmental processes in plant. The oxidoreductase enzymes also play crucial role in electron transport of a wide variety of chemical reactions in the plant cell.

**Table 2:** Gene transcripts differentially upregulated in upland VS16 as compared to the lowlands AP13 and K5 switchgrass with more than two-fold change (q-value ≤0.01)

Rice homolog function description	FPKM_ VS16	FPKM_ lowland	Average Fold change
AP2 domain-containing protein	77.5	345.0	4.4
auxin-repressed protein	55.9	341.3	53.9
BTBN22 - Bric-a-Brac, Tramtrack, Broad Complex BTB domain with non-phototropic hypocotyl 3 NPH3 and coiled-coil domains	80.5	250.7	3.3
catalase isozyme A	30.6	330.8	30.5
FAD-binding and arabino-lactone oxidase domains containing protein	60.2	414.5	2081.4
glycine rich protein family protein	202.9	554.0	2.8
heavy-metal-associated domain-containing protein	60.4	238.4	4.4
helix-loop-helix DNA-binding domain containing protein	436.7	989.7	2.3
HVA22	60.6	267.9	4.6
late embryogenesis abundant group 1	0.1	671.3	4590.7
LTPL29 - Protease inhibitor/seed storage/LTP family protein precursor	123.9	853.1	7.8
magnesium-chelatase	80.0	202.0	2.6
methyltransferase	0.5	211.7	580.0
NADP-dependent oxidoreductase	142.6	375.6	2.7
PHD-finger family protein	3.1	327.5	109.5
phytosulfokines precursor	52.7	292.6	5.7
POT family protein	223.6	881.8	6.5
ribonuclease T2 family domain-containing protein	143.3	857.3	88.4
S-adenosylmethionine synthetase	78.6	245.7	3.3
SRC2 protein	114.4	310.0	2.8
universal stress protein domain containing protein	148.2	327.2	2.2
wound induced protein	128.6	397.2	3.3

As light is the prime source of energy for plant growth and morphogenesis, variation in light harvesting may arise from morphological and physiological differences in plants. This result showed that the southern-adapted lowlands seemed more efficient in photosynthesis than the uplands. As gene expression is the result of environmental and developmental changes, transcriptome divergence between the two ecotypes implies adaptive phenotypic selection [28], and the pattern underlying the adaptive evolution occurred between the two ecotypes to adapt to their current habitat. The lowlands are adapted to the southern continental USA with relatively longer growing season and high solar radiation. It is evident that the lowland ecotypes have maintained efficient light perception and carbon reduction mechanism, which ultimately transformed into high biomass accumulation. On the other hand, the uplands are adapted to the shorter growing season of the upper latitudes and have reduced plant stature, which may be due to the low light reception and carbon reduction efficiency. With the hypothetical south-to-north migration path of switchgrass [29], we speculate that the upland ecotypes were derived from the lowland ecotypes through loss of function adaptive evolution.

### Gene Ontology (GO) enrichment of DEGs

**Fig. 3:**
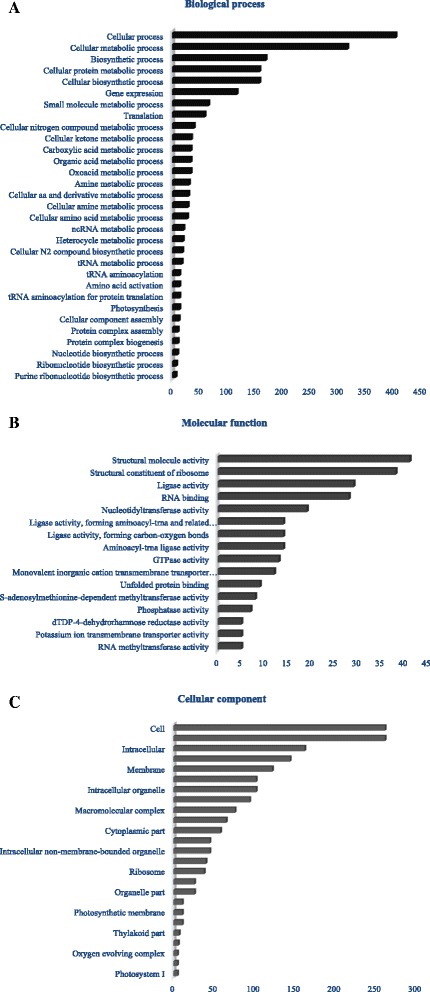
The Gene Ontology (GO) terms in the three GO domains (**a** biological process, **b** molecular function and **c** cellular process) that are significantly (q ≤ 0.05) overrepresented in the lowland compared to the custom annotated reference switchgrass genome (background) as analyzed using AgriGO and slim-GO

**Fig. 4:**
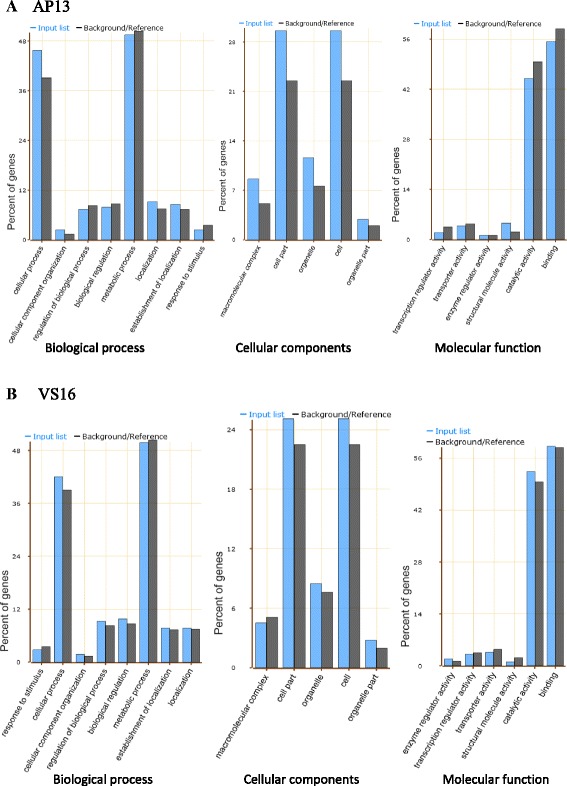
Functional category of Gene Ontology (GO) of AP13 and K5 (**a**) and VS16 (**b**) gene transcripts compared to the switchgrass reference genome (*P. virgatum* v 1.1). *Blue* colored bars represent the input list and the *black* colored bars represent percent of reference/background genes against the whole slimGO terms

Among the significant biological processes, cellular process, and cellular component (Fig. 3) organization and localization were significantly higher (p ≤ 0.05) in the AP13 as compared to the reference (Fig. 4a). The percentage of cellular components such as macromolecular complex, cell and organelle were higher in AP13 than the proportion in the reference annotated pool. The proportion of most of the molecular functions, except structural molecular activity, was significantly lower in AP13 than the reference pool. None of the annotations of the VS16 was significantly different from the reference pool in proportion. However, cellular process and regulation of biological process in the biological process; cell, cell part, organelle and organelle part in the cellular component; and enzymatic activity and catalytic activity among the molecular function were higher in proportion but not significantly different from the reference (Fig. 4b). This implies that the variation in ecotype is not in a particular set of gene families but in the overall level of expression across a wide range of gene families.

### Identification of SNPs and Indels between genotypes and ecotypes

**Fig. 5:**
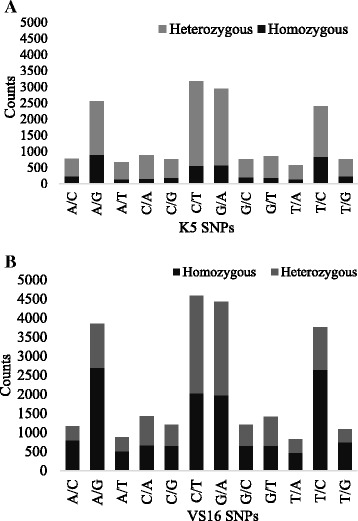
Number of SNPs detected for K5 (**a**) and VS16 (**b**) against the reference AP13 alleles at ≥100x coverage and ≥5% minor allele frequency. Counts for all possible allele combinations of the query and the reference (AP13) were graphed for homozygous and heterozygous SNPs

Homozygous and heterozygous SNPs discovered for VS16 and K5 against the reference AP13 genome were presented in Fig. 5. A total of 25,894 and 16,979 SNPs were discovered at ≥100x coverage and ≥5% minor allele frequency for VS16 and K5, respectively (Additional file 4: Table S3). The 8,915 SNPs that were different between VS16 and K5 may reflect the ecotype variation for the polymorphism. For VS16, except for C/A, C/T, G/A, and G/T, the number of homozygous SNPs are larger in number than the heterozygous SNPs. On the contrary, for K5, heterozygous SNPs are larger in number than the homozygous SNPs for each of the transition as well as transversion SNPs. Among the 12 types of SNPs assessed, based on the allele combinations, it was observed that C/T, G/A, A/G and T/C are the abundant SNPs detected for both K5 and VS16 individually against the reference genome (Fig. 5). In both the VS16 and K5, the SNPs of A/T or T/A were less represented. This implies that transition mutations within pyrimidine (C, T) and purine (A, G) are more prevalent than the transversion mutation, which converts pyrimidine bases into purines or the vice versa.

**Fig. 6:**
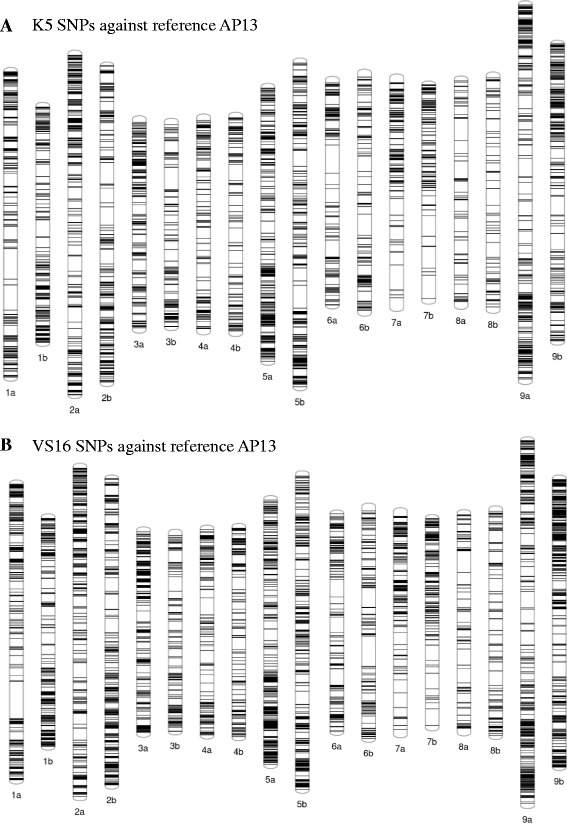
Distribution at base-pair locations of SNP variants called from RNA-Seq transcripts for K5 and VS16 against the reference AP13 genome sequence. The distribution is depicted on nine homeologous pseudomolecules of switchgrass genome (1–9 **a** and **b**)

Among the SNPs identified, 66% (11,166/16,979) of K5 and 64% (16,625/25,894) of VS16 were mapped on the reference AP13 pseudomolecules. The distribution of SNPs detected for K5 and VS16 were visualized on the reference genome’s pseudomolecules (Fig. 6). The overall distribution indicated that there is high density of SNPs in distal (telomeric) regions than the proximal end of the chromosomes. There are uneven distributions among the chromosome arms indicating some part of the genome have high recombination events than the others. There are also several gaps on each of the chromosomes, which are potential centromeres. In chromosome 7a and 7b, one arm has higher density than the other. On the other hand, chromosome 8a and 8b, had the least SNPs overall, distributed across both arms. From the linkage mapping it was not possible to form chromosome 7a and the probable reason was predicted as either high homozygosity or aneuploidy [10]. From this low SNP density we could substantiate that high homozygosity is more relevant for the disappearance of the linkage group using the PCR-based and DArT markers.

Next generation sequencing (NGS) technology has been used to detect SNPs in crops such as wheat (*Triticum aestivum*), barley (*Hordeum vulgare*) [30], cotton (*Gossypium hirsutum*) [31], rice (*O. sativa*) [32], soybean (*Glycine max*) [33], potato (*Solanum tuberosum* L.) [34] and sorghum (*Sorghum bicolor*) [35, 36]. DNA sequence changes in the non-coding region of the genome may disrupt functional cis-regulatory elements that control transcription and leads to change in transcription levels, while mutation in the protein coding regions leads to altered form of protein [37]. These SNPs are from gene sequences and indicate allelic variations among genotypes and/or between ecotypes for the genes expressed at different levels.

All of the SNPs detected on the unanchored contigs in or near the gene sequences were screened at 100x coverage and 5% minor allele frequency. In addition to the SNPs, Indel polymorphism for K5 and VS16 identified relative to the reference genome were presented in Additional file 5: Table S4. A total of 143 and 439 Indels were observed for VS16 and K5. Again, high Indels were detected between the lowland AP13 and the upland VS16, implying allelic differences in genes transcribed between the two ecotypes.

### Conserved simple sequence repeats (SSRs) among the genotypes

With the aim of developing SSR markers that are polymorphic among genotypes and ecotypes, a de novo assembly was done for the K5 and VS16 transcripts. Consequently, 1,295 EST-SSRs that are conserved among the two and three genotypes were identified (Additional file 4: Table S3). Among the three genotypes, 312 genic SSRs were detected, while the remaining 983 were between a pair of genotypes. The conserved SSRs were also polymorphic among at least two of the three genotypes. Genic SSRs were developed for switchgrass from different genotypes [38, 39] of which some were used for genetic linkage mapping of the switchgrass genome [10, 40–42].

## Conclusions

In the present study, comparison of expression profiles between the lowland and the upland ecotypes of switchgrass during active growing stage disclosed a clear difference in photosynthetic efficiency between the two ecotypes. A genome-wide differential upregulation of chloroplast precursors and photosystem proteins in the lowland compared to the upland ecotype revealed that the lowland ecotype is more efficient in photosynthesis than the upland ecotype. The GO annotation of the transcripts indicated that lowland ecotype has significantly higher photosynthesis machinery for efficient light perception and more carbon fixation. A number of SNP and conserved SSR markers that were polymorphic between genotypes and ecotypes were detected throughout the genome. This discovery would be a useful resource for trait mapping and gene transfer across ecotypes to significantly facilitate switchgrass breeding for increased biomass yield and biofuel conversion.

## Methods

### Plant materials and experimental design

Two genotypes representing the tetraploid lowland switchgrass, AP13 and K5, and a genotype representing the upland ecotype, VS16, were selected for the experiment. The three selected genotypes were clonally propagated by splitting tillers in the greenhouse to plant three replicates. The growth chamber experiment was arranged in a randomized complete block design consisted of three replicates. For each replicate, there were three plants of each clone in a pot. The growth chamber was maintained at 29/22 °C day/night temperatures, and a 16-h photoperiod, with a photon flux density of 150 to 200 μmol m^−2^ s^−1^.

### RNA extraction and library preparation

Total RNA was isolated from fully expanded young leaf tissues at third elongation (E3) [43] stage. The RNA was extracted using TRIzol® Reagent (total RNA isolation reagent) following the manufacturer’s instructions (Life Technologies, Grand Island, NY, USA) as previously described [44]. The RNA was pooled from three independent pots of each genotype and for each of the three biological replicates. RNA was eluted in RNase-free water and quantified using a NanoDrop1000 spectrophotometer (Thermo Scientific, DE, USA) and RNA integrity was evaluated with RNA6000 n Assay using the Agilent 2100 Bioanalyzer™ (Agilent Technologies, Palo Alto, CA, USA) according to manufacturer’s instructions. The TruSeq stranded total RNA library preparation kit (Illumina, San Diego, CA, USA) was used for cDNA libraries construction as detailed in Serba et al. [44]. In brief, polyA containing messenger RNA was first purified from total RNA using poly-T oligo-attached magnetic beads and then chemically fragmented and primed with random hexamer priming for single-stranded cDNA synthesis. A second cDNA strand was synthesized as a replacement strand to the RNA to create double-stranded cDNA that was ready for TruSeq library construction. The short overhanging ds-cDNA fragments were converted to blunt ends using T4 DNA polymerase. Then, the 3’ blunt ends were adenylated and ligated to multiple sequencing adapters for hybridization into a flow-cell. Finally, RNA libraries were built by PCR amplification to enrich RNA fragments with adapter molecules by PCR primers annealed to the end of the adapters. The RNA libraries were quantified using qPCR, according to the Illumina Sequencing Library qPCR Quantification Guide. Normalization of the indexed libraries to 10 nM was conducted. Finally, quantification was carried out using the Agilent Technologies 2100 Bioanalyzer and pooled for sequencing.

### RNA-Seq, preprocessing and mapping

Paired-end (2 × 100 bp) of the cDNA libraries were sequenced using Illumina TruSeq™ sequencing on the HiSeq™ 2000 platform (Illumina). Quality evaluation and trimming was as described in Serba et al. [44]. Briefly, in-house program was used to trim out low-quality bases (<Q15) from both the 5’- and 3’- ends of the reads to ensure two or more consecutive bases obtained a Phred score of 30 (99.90% base call accuracy) or more. The trimmed reads were aligned onto the switchgrass reference genome sequence (http://www.phytozome.net/panicumvirgatum v1.1) using TopHat2 [45] with underlying mapping with Bowtie2 [46], allowing multi-mapped reads and a maximum of two mismatches per read with other default settings. Transcripts were quantified with Cufflinks and comparison of normalized transcript counts between the genotypes was done using Cuffdiff [47–50] in FPKM expression level [51]. Differential expression between samples was assessed at 5% false discovery rate (FDR). Gene Ontology (GO) [52, 53] analysis of differentially expressed genes was performed using the Singular Enrichment Analysis (SEA) tool of the AgriGO (http://bioinfo.cau.edu.cn/agriGO) [54] with annotations derived from the switchgrass (*Panicum virgatum* v1.1) genome sequence.

### Gene expression analysis

Illumina reads were mapped on the switchgrass reference genome (*P. virgatum* v1.1) using the default parameters. PCR duplicates were removed with Samtools rmdup option [55] and an annotation-guided read alignment was performed with Cufflinks v2.1.1 to reconstruct transcripts and estimate transcript abundance in units of FPKM. Regions with FPKM values higher than zero were considered as expressed [56]. Based on the semi-quantitative measurement of the FPKM scale defined by Hansey and collaborators, expressed genes can be divided in three classes [57]: genes with a FPKM value below 5 are low-expressed, genes with a FPKM value greater or equal to 5 and less than or equal to 200 are medium-expressed, and genes with a FPKM value greater than 200 are highly-expressed.

### Ecotype and genotype comparisons

Three-way comparisons were conducted among the two lowland and an upland genotypes (Fig. 2). The gene expression of the upland genotype VS16 was compared separately with the AP13 and K5. Also the two lowland genotypes, AP13 and K5, were compared to find out genotypic differences within the same ecotype. The differentially expressed gene sets for each of the three genotypes were depicted in a Venn diagram using a web-based method called InteractiVenn [58].

### SSRs and SNP markers development

To identify conserved SSRs among the genotypes, a de novo assembly was conducted for the K5 and VS16 transcripts. These new transcripts and the annotated transcripts from the reference genome (AP13) were run through RepeatMasker 4.0 [59]. Then, the de novo assembled transcripts of K5 and VS16 were BLASTn against the reference transcripts (AP13) to find homologs pairs and triplets that contained the same SSRs.

Transcripts mapped to the reference genome were converted from SAM to BAM and sorted for variant discovery. Then, SNP and indels based on the mapped reads were called using SAMtools mpileup v0.1.7a (http://samtools.sourceforge.net) [55] and Bcftools. Custom Perl scripts were used to reformat the original output and filter the SNPs based on coverage and probability. Another Perl script was used to identify in which gene each SNP was found and where it was located relative to that gene sequence (exon or intron, upstream or downstream). The variants were called separately for K5 and VS16 against the reference (AP13). The SNPs were first called for at least 5% coverage. Next, the SNPs were refined for over 100x coverage and at least 5% minor allele frequency. The SNPs discovered on unanchored contigs were checked for their location with respect to exon sequences of genes. Similarly, Indels and SSRs were detected using MIcroSAtellite identification tool (MISA v1.0) [60]. Minimum unit size cutoffs of eight for a di-, six for a tri-, and four for tetra-, penta-, and hexanucleotide repeats were used to report SSRs. A maximum distance of 100 bp was allowed between two SSRs. A web based PhenoGram [61] was used to visualize the distribution of the SNP loci across the switchgrass pseudomolecules, each line representing a SNP locus with its base pair coordinate.

### Availability of supporting data

Supporting data are included as a Additional file 1: Table S1, Additional file 2: Table S2, Additional file 3: Figure S1, Additional file 4: Table S3 and Additional file 5: Table S4.

## Additional files

Additional file 1: Table S1. Analysis of variance for the number of RNA-Seq clean reads for AP13, K5 and VS16 switchgrass genotypes across three replicates (XLSX 8707 kb)
Additional file 2: Table S2. Gene expression (upregulation) among AP13 (LL), K5 (LL), and VS16 (UL) switchgrass genotypes (LL = lowland ecotype, UP = upland ecotype, 4X = tetraploid). (XLSX 2247 kb)
Additional file 3: Figure S1. KEGG map for photosynthesis populated with transcripts coding for chloroplast precursors and specific enzymes in the photosynthetic pathway. (DOCX 375 kb)
Additional file 4: Table S3. SNPs in or near genes detected for K5 and VS16 against AP13 at ≥100x coverage and 5% minor allele frequency. (XLSX 91 kb)
Additional file 5: Table S4. Polymorphic conserved simple sequence repeats (SSR) markers detected among the two lowland and an upland genotypes. (DOCX 15 kb)

## Abbreviations

AP13: Alamo Plant 13
ATP: Adenosine triphosphate
DArT: Diversity arrays technology
DEGs: Differentially expressed genes
FPKM: Fragments Per Kilobase of transcripts per million Mapped reads
K5: Kanlow genotype 5
KEGG: Kyoto Encyclopedia of Genes and Genomes
KOG: EuKaryotic Orthologous Groups of proteins
NADP: Nicotinamide adenine dinucleotide phosphate
PANTHER: Protein ANalysis THrough Evolutionary Relationships
PFAM: Protein FAMiliy
slim-GO: Gene Ontology (cut-down version)
SNP: Single nucleotide polymorphism
SSR: Simple sequence repeat
VS16: Summer Plant 16
