# Supplementary figures and images for: Transcriptome analysis in switchgrass discloses ecotype difference in photosynthetic efficiency
Source: BMC Genomics. 2016 Dec 16;17:1040. doi: 10.1186/s12864-016-3377-8 (PMC5162099; doi:10.1186/s12864-016-3377-8)

Supplementary Figure 1


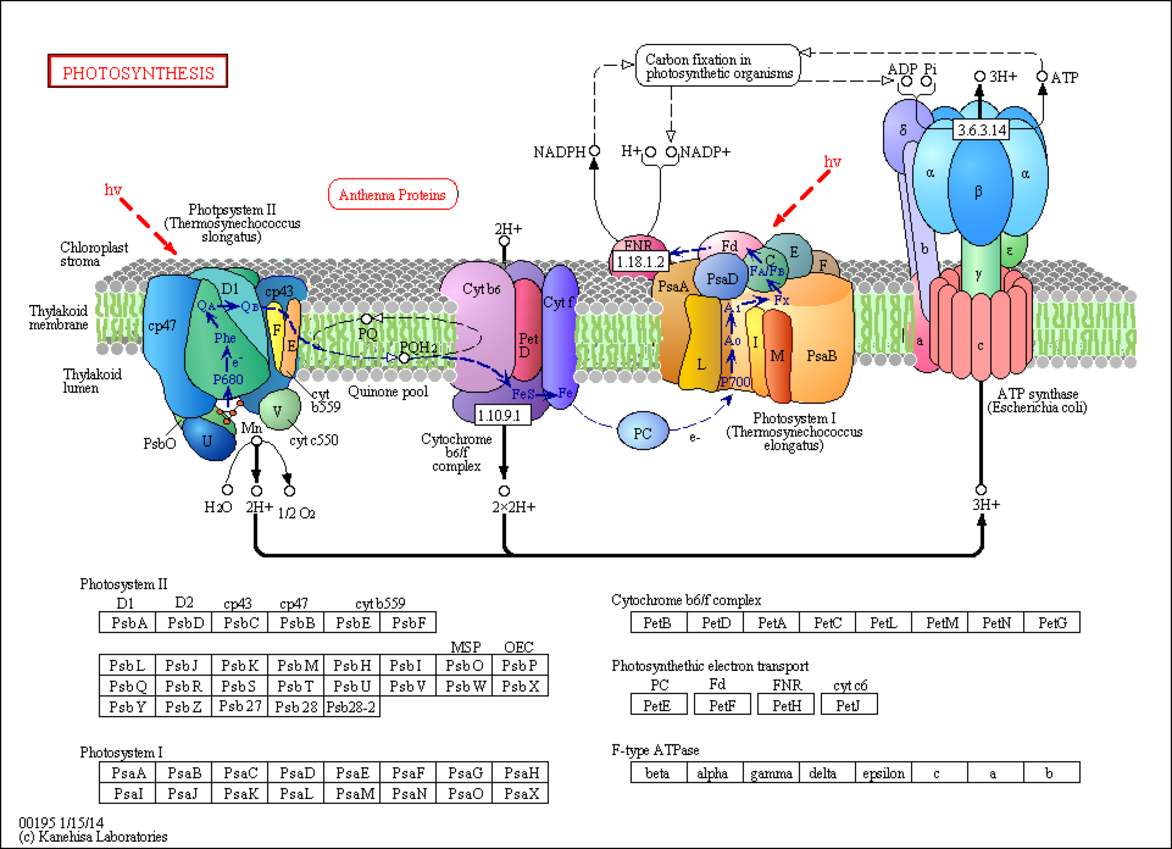

Supplement: Additional file 3: Figure S1. — KEGG map for photosynthesis populated with transcripts coding for chloroplast precursors and specific enzymes in the photosynthetic pathway. (DOCX 375 kb) [file 12864_2016_3377_MOESM3_ESM.docx]
