# Supplementary material for: Transcriptome analysis in switchgrass discloses ecotype difference in photosynthetic efficiency
Source: BMC Genomics. 2016 Dec 16;17:1040. doi: 10.1186/s12864-016-3377-8 (PMC5162099; doi:10.1186/s12864-016-3377-8)
Supplement: Additional file 5: Table S4. — Polymorphic conserved simple sequence repeats (SSR) markers detected among the two lowland and an upland genotypes. (DOCX 15 kb) [file 12864_2016_3377_MOESM5_ESM.docx]

**Additional file 5: Table S1**

| **Source** | **DF** | **Mean Square** | | **P-value (total)** | **p-value (mapped)** |
| --- | --- | --- | --- | --- | --- |
|  |  | **Total clean reads** | **Total mapped clean reads** |  |  |
| Replication | 2 | 2.26 | 3.63 | 0.78 | 0.68 |
| Genotype | 2 | 59.34 | 8.02 | 0.06 | 0.46 |
| Error | 4 | 2.97 | 2.90 |  |  |
| LSD (0.05) |  | 6.73 | 6.58 |  |  |
| CV (%) |  | 10.08 | 12.46 |  |  |
| R^2^ |  | 0.78 | 0.41 |  |  |

DF=degree of freedom
